# A nutrition-focused review of the interventions in US-living Latino communities with type II diabetes

**DOI:** 10.3389/fnut.2024.1418683

**Published:** 2024-09-18

**Authors:** Mélanie Guirette, Magdalena Sevilla-Gonzalez, Maureen Balaguera, Augusto Enrique Caballero

**Affiliations:** ^1^Friedman School of Nutrition Science and Policy, Department of Nutrition Epidemiology and Data Science, Tufts University, Boston, MA, United States; ^2^Clinical and Translational Epidemiology Unit, Mongan Institute, Department of Medicine, Massachusetts General Hospital, Boston, MA, United States; ^3^Department of Medicine, Harvard Medical School, Boston, MA, United States; ^4^Programs in Metabolism and Medical & Population Genetics, The Broad Institute of MIT and Harvard, Cambridge, MA, United States; ^5^Southern Jamaica Plain Health Center, Brigham and Women’s Hospital and Harvard Medical School’s Teaching Hospital, Boston, MA, United States; ^6^Brigham and Women’s Hospital and Harvard Medical School, Boston, MA, United States

**Keywords:** Latinos, type 2 diabetes, cultural relativism, medical nutrition therapy, dietary assessment methodologies

## Abstract

Type 2 diabetes (T2D) is a chronic, debilitating disease that disproportionally affects the Hispanic/Latino community residing in the United States. Optimal nutrition therapy is fundamental to the proper management of T2D and must be culturally adapted to facilitate permanent behavior change in this population. This review selected and assessed the nutrition components of interventions aimed to improve T2D outcomes in US-based Latinos/Hispanics, published from 2002 to 2023. An overview of the participant characteristics, nutrition intervention, and dietary assessment and outcomes is included. Nutrition interventions in this community benefit from the inclusion of bicultural registered dietitian nutritionist (RDNs) to assure the counseling team promotes culturally tailored nutrition recommendations based on current dietary guidelines. Nutrition assessment and outcomes should be captured with the use of validated dietary assessment tools and dietary quality indices appropriate to their target population. Standardizing these practices will facilitate intervention comparability and replicability and ultimately better target the needs of this community.

## 1 Introduction

Culturally appropriate dietary recommendations are particularly important for the Latino community in the United States, which is both disproportionally affected by T2D and carries rich culinary traditions that vary according to their country of origin. Indeed, the prevalence of diagnosed diabetes among US-living adults of Hispanic origin is of 11.7% (compared to 6.9% in their non-Hispanic White counterparts); and that prevalence varies depending on the Hispanic ethnicity subgroup (Puerto Rican at 13.3%, Mexican at 11%, Dominican at 9.4%, and Cuban at 9.0%) (1). Furthermore, the Hispanic Community Health Study/Study of Latinos (HCHS/SOL) landmark study reported a mere 1.7% of participants met the Ideal diet criteria for cardiovascular health (2). Although common staple foods can be found in most Latin American countries, each nation has its own culinary traditions that are upheld after these groups migrate to the United States (3). There is consequently an urgent need for medical nutrition therapy (MNT) tailored to the cultural background of each patient to effectively address the disproportionate health disparities within the US Latino community.

Optimal nutrition therapy is one of the cornerstones of type 2 diabetes (T2D) prevention and management. Indeed, strong evidence supports MNT carried out by a registered dietitian nutritionists (RDNs) can effectively reduce hemoglobin A1c (HbA1c) by 2.0% in patients with T2D (4). The American Diabetes Association’s (ADA) consensus recommendation on dietary patterns for T2D management include emphasizing consumption of non-starchy vegetables, minimizing added sugars and refined grains, and choosing whole foods over highly processed foods whenever possible. However, this is not a “one size fits all approach,” as dietary habits and food choices are influenced by many factors outside of health management, such as cultural and personal preferences, socioeconomic status, the food environment, etc. (5). Consequently, successful diabetes self-management education and support (DSME/S) should tailor its nutrition component to the individual’s environment and cultural background.

Tailored nutrition interventions are necessary for an overall successful DSME/S, however the process and impact of the current interventions remain understudied in this population. Reviews on interventions for US-based Latinos with T2D have studied the effectiveness of their cultural components (6), the use of community health workers (CHW) (7), the emotional well-being in Latinos (8), and even “innovative approaches” on T2D disease progression (9); however, no review has focused on assessing the interventions’ nutrition component and dietary outcomes. Consequently, we conducted a scoping review of nutrition interventions targeting Hispanic/Latino communities in the United States with T2D, aiming to identify key characteristics that are either commonly shared or notably absent in these interventions.

## 2 Methods

### 2.1 Literature search

We comprehensively searched five electronic databases including PubMed, EMBASE, Cochrane, CINAHL, ERIC, and PsycINFO using combinations of the following keywords: (nutrition OR nutrition intervention OR nutrition trial OR diet OR diet intervention OR diet trial OR nutrition program OR diet program OR medical nutrition therapy) AND (Hispanic OR Latino OR Mexican OR Puerto Rican OR Dominican OR Central American OR South American OR Cuban) AND (type 2 diabetes OR type 2 diabetic OR diabetes OR diabetic OR T2DM OR T2D). We further applied the following filters: clinical trials and controlled clinical trials for article type, English for article language, Humans for study species, and publication date within the last 20 years (2002–2023).

Article titles were combined, and duplicates were removed using Endnote (version 20.4) before transferring titles to Rayyan where independent title and abstract screening took place by two reviewers (MG and MS-G). The reviewers discussed conflicted articles and the final list was re-transferred to EndNote where full text articles were stored for handsearching individual bibliographies and developing the study’s reference list. Studies were excluded if the study population was not diagnosed with T2D or exclusively self-identified Hispanic/Latinos living in the US. We included all primary, secondary, and pilot studies with nutrition/diet related outcomes. We then further excluded if the intervention protocol lacked a well-documented nutrition intervention or if the measurements captured by the intervention lacked any sort of nutrition/diet related outcome. We reviewed the references of the articles selected for full text screening for inclusion.

### 2.2 Data extraction

Data collected from each article included information pertinent to the intervention’s participants, frameworks, and outcomes. Specifically, participant location (both geographical and intervention environment), mean age, sex, education, income, health insurance status, and acculturation metrics were extracted from the articles’ study characteristics tables. Intervention information included sample size, intervention period and number of sessions, background on the counselor delivering the intervention, and control group treatment (if any). We also reported if the researchers specified their intervention as a pilot study, the use of a particular framework or model for targeted behavior change, the number/h and description of nutrition education sessions, and the specific culturally tailored components of the interventions. Finally, the dietary outcomes, the specific tools used to measure those dietary outcomes, and changes in HbA1c and/or weight were also captured in this review. We reported both significant and non-significant changes in HbA1c and weight due to the inclusion of both pilot studies and full-scale interventions, as well as the fact that clinically significant outcomes do not always overlap with statistical significance (10).

## 3 Results

### 3.1 Study selection

**Figure 1:**
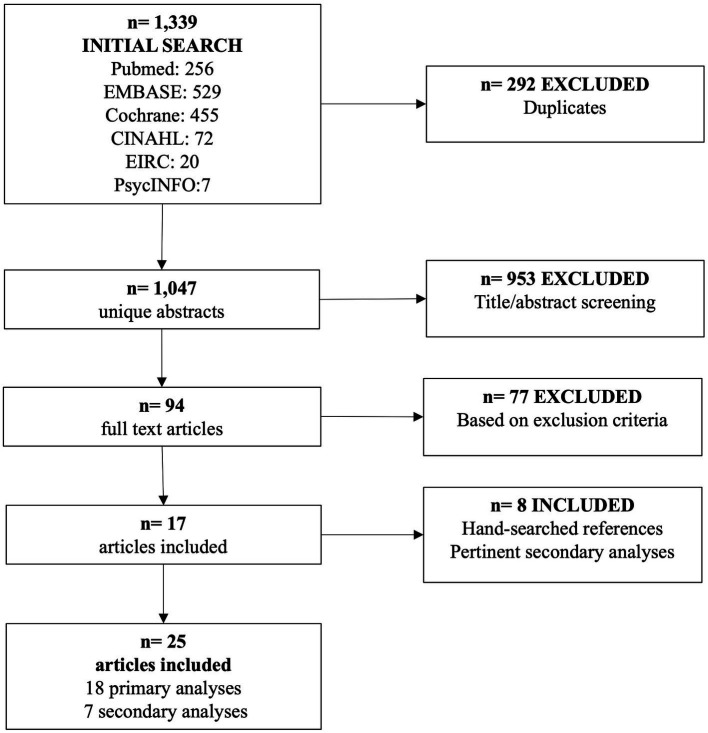
Flow chart of literature search.

The search and study selection process are described in Figure 1. The combined search of the five databases yielded 1,339 abstracts, 292 of which were identified as duplicates and 953 were further excluded by the title/abstract screening. Out of 94 articles assessed by full text screening, 17 fit our inclusion criteria (11–27). We then included an additional seven articles (28–34) that were referenced by the included articles and one additional secondary analysis (35) from our included articles with interesting nutrition/diet related outcomes. A total of 25 articles (18 primary analyses and seven secondary analyses) are included in this review. Of note, at least 47 studies assessed during the full text screenings of articles found in the database and reference searches had to be excluded because no dietary outcome of any kind was captured by the intervention.

### 3.2 Participant characteristics

A summary of the general reported characteristics of participants in our selected interventions is reported in Table 1. Sample sizes ranged from 21 to 300 participants, mean ages ranged from 49.3 to 62.6 years, and female participants ranged from 55 to 100% of the participants. Most of the studies were carried out in participants with Mexican ethnical origin living predominantly in California, Texas, and Chicago; followed by participants with Puerto Rican and Dominican ethnical origins mostly living in Connecticut and Massachusetts. Nine studies reported at least 88% of their participants were born outside of the US. A part from *¡Viva Bien!*, Spanish was the primary or home language for 61–97% of the participants in the 12 studies that reported this metric. Six studies measured acculturation, where three reported an average score ranged from 0 to 1.6 out of five (36) and the other three reported 48–100% of the participants with a “low” acculturation score. In terms of education, only four out of the 16 studies capturing this metric reported most participants attaining 12 or more years of education. In terms of income, only two studies reported most of their participants had an annual household income above $20,000/year. Finally, participants received the intervention exclusively in a community setting (churches and libraries) for two studies, exclusively in outpatient or health clinics in 10 studies, or a mixture of the two in 4 studies. Two studies carried out part of their interventions in participant homes.

**Table 1:** General reported characteristics of participants.

Intervention name^1^	Location		Sample size^2^	Mean age (SD)	% Female	Hispanic origins	Education	Income	Insured (%)	Born outside of US (%)/ time in US	Spanish main language^3^	Acculturation
	Recruitment	Recruitment/intervention										
*Imagínate una Buena Salud*	South Lawndale neighborhood, Chicago, IL	Churches (2)	100	53.7 (12)	81	95% Mexican	54% ≤ 8th grd	86% < $30 K/yr	49	92%	71	
IMB-DSC	Hartford, CT	OCC (1)	91	57.7 (15.2)	74	PR	59% ≤ 8th grd		89	25 yr	87	
*EnForma-Diabetes*	Central North Carolina	OCC (1)	21	52.3 (8.2)	57	71% Mexican 4% CA, 2% SA	71% < HS	67% < $20 K/yr	33	100%		100% low
Cariño	Miami-Dade County, FL	OCCs (2)	300	55.3 (9.4)	55	38% Cuban	42% < 12 yr	39% ≤ 1,000/mo	19			48% low
*DIALBEST*	Hartford, CT	OCC (1); Subject Home	211	56.3 (11.8)	74	PR & DR	48% ≤ 8th grd	79% ≤ 1,000/mo			65	
MATCH	Chicago, IL	Subject Home; Rush University Medical Center	144	53.7 (12.2)	67	Mexican	57% ≤ 6 yr				90	1.6^4^
28447545	Hartford, CT		157	53.5 (9)	65	Mexican	50% ≤ 5th grd	77% < $25 K/yr		28 yr	71	65% low
26957533	Central North Carolina	Clinics and churches (6)	92	49.4 (11.3)	59	80% Mexican	75% > 12 yr	53% < $10 K/yr	29	97%	97	
28503930	San Bernardino County, CA	Community Clinics & Church	32	53.1 (14.7)	78	80% Mexican	40% ≤ 5th grd		20	24mo		
*Unidas por la Vida*	CA	Health Center	89	52.7 (6.9)	100	Mexican	83% < HS	94% < $30 K/yr		95%	73	
*Amigos en Salud*	Los Angeles, CA	Family Health Centers (3)	189	50.0 (11.9)	64		61% < 6th grd	53% < 25 K/yr			96	
Si, Yo Puedo Controlar Mi Diabetes!	Hidalgo County & Starr County, TX	Churches and Libraries	139	61.7 (14.0)	74		73% ≤ HS	71% ≤ $20 K/yr	50		97	50% scored 0†
15946117	Western Massachusetts, MA	Community Health Center	25	62.6 (8.6)	80	PR	74% ≤ 8th grd	100% ≤ $20 K/yr	100			
La Diabetes y La Unión Familiar	Santa Cruz County, AZ	Subject Home; “Central Location”	72									
21462725	Star County, Texas	Not reported	63	49.3 (8.4)	69	Mexican					61	1.2^5^
*¡Viva Bien!*	Denver, CO	OCCs (9)	280	57.2 (14.2)	100	Mostly Mexican	24% < 12th grd	43% < $30 K/yr			16	
*En Balance*	San Bernardino and Riverside Counties, CA	Loma Linda University	31	54 (10.7)	58	Mexican majority PR and DR minority	39% ≤ 5th grd			94%	90	
*Latinos En Control*	Springfield, MA	Community Health Centers (5)	252		77	87% PR	56% ≤ 8th grd	55% < $10 K/yr	98	88%		

1 Intervention referred to by their nickname or study PMID when nickname was not available.

2 Reported in Study Characteristics tables of included articles.

3 Described as either “Primary” language or “language spoke at home”.

4 Based on a scale developed by Marin et al.

5 Range 1–4 with 4 = high acculturation.

Empty cells indicate information was not reported by the researchers in article nor previously published protocol. CA, Central America; DR, Dominican Republic; grd, grade; HS, High School; mo, month(s); OCC, Outpatient Care Clinic; PR, Puerto Rico(an); SA, South America; yr, year(s).

### 3.3 Intervention characteristics

Key characteristics of the 25 interventions are summarized in Table 2. Eight studies labeled themselves as pilot studies. Intervention periods ranged from 1.2 to 24 months, and five to 36 sessions. Most of the interventions were carried out in 3–6 months with 10–12 sessions. In terms of control groups, three did not receive any treatment at the time of the intervention; two exclusively received education material, seven received either DSME/S or usual care, and three received a combination of education material with either DSME/S or usual care. Three studies did not report having a control group (two of them being pilot studies).

**Table 2:** Intervention characteristics.^1^

Intervention name	Pilot Study?	Intervention period (months)	# Nutrition sessions	Counselor	Control	Sessions	Intervention framework	Culturally tailored component
*Imagínate una Buena Salud*	Y	2		bilingual, lay leaders	DSME only	8	Self Determination Theory	Healthy preparation of traditional Mexican recipes
IMB-DSC		3		bilingual, PR MA	usual care		The Information-Motivation-Behavioral Skills (IMB) model	Culturally tailored, individualized meal plan booklets Culturally familiar foods Intervention content available in Spanish (Puerto Rican dialect) and English
*EnForma-Diabetes*	Y	2		RD	none	4	Mediterranean style dietary intervention	Cultural and linguistic refinements for Hispanic Americans with T2D were addressed through interviews with HA food preparers and focus groups with HA patients diagnosed with T2D Spanish language version of a Mediterranean (Med)-style dietary intervention so that the dietary recommendations align with the cultural and social needs of Hispanic Americans
Cariño		12	6	CHW	usual care + Mailed Education Material	10	multilevel CHW intervention	Culturally relevant resources, manuals, and tool kits
*DIALBEST*		12	9	Bilingual/bicultural, RD & MA	usual care	17	Behavioral Change Theory	Culturally and health literacy appropriate counseling
MATCH		24		bilingual Mexican Americans, CHWs	bilingual newsletter	36	Self-Management & Social Cognitive Theories by AADE	CHWs taught in the participant’s preferred language and used culturally appropriate examples or metaphors
28447545		4		RN (CDE) & *promotora*	waitlist	12	Self-management & Social Support	Use of Spanish language Dietary and physical activity preferences common in the border region Social support congruent with the traditional Mexican cultural values of *simpatía* (kindness, politeness), *respeto* (respect), and *personalismo* (formal friendliness)
26957533		9	~ 1.5	bilingual and bicultural, RNs	General health promotion information & 2 DSME sessions	10	DSMEP and NDEP	Modules tailored to low-literacy needs and integrated cultural beliefs and values Use of modified ethnic foods and recipes and culturally relevant activities Family-focused intervention incorporated cultural values (*familismo*)
28503930	Y	6	2	Educator/Facilitator	usual care	5	DSMEP	All sessions were taught in Spanish Culturally aligned menus of high-fiber, low-fat foods
*Unidas por la Vida*	Y	4		lifestyle community coach	Mailed Education Material	12	Diabetes Prevention Program’s (DPP) Lifestyle Change Program	Use of modified recipes from a Hispanic/Latino diet
*Amigos en Salud*		6		bilingual Hispanic CHWs (3)	usual care + Education Material	11.3	Transtheoretical (stages of change) model	Used culturally appropriate educational materials
Si, Yo Puedo Controlar Mi Diabetes!	Y	1.2		bilingual, RN & RD	waitlist	5	Social cognitive theory and the Self-Regulation Theory	Used telenovela framework
15946117	Y	2.2		bilingual diabetes RN, RD, lay leader	Received labs at assessments	14	Social Cognitive Theory	Attendance by family members to elicit home-based support/approval Teaching of diabetes-related knowledge through culturally popular activities (e.g., a soap opera) Teaching/counseling about dietary change by modifying ethnic foods and recipes Inclusion of opportunities for socializing (e.g., “coffee time” for informal conversation prior to beginning of each session) Delivery of intervention in the preferred language (English/Spanish)
La Diabetes y La Unión Familiar		3		*promotoras* (4)	none	10	Social Learning Theory	Encouraged family members to collectively set health-behavior goals, to overcome obstacles hindering healthy behaviors, and to develop a plan to sustain behavior changes All training, intervention and evaluation materials and activities were produced and delivered in Spanish
21462725	Y	6		bilingual, Mexican-American RD, NCM (CDE), and CHW	DSME only	10	DSME + Nurse Case Manager	Culturally competent in language, diet, social emphasis, family participation, and incorporation of cultural health beliefs typical Incorporated Mexican American dietary preferences into dietary recommendations Food demonstrations based on healthy adaptations of favorite Mexican American recipes Subject identified a family member, preferably a spouse or first-degree relative, who agreed to participate as a support person
*¡Viva Bien!*		12	1		usual care	2.5d retreat +36	MLP EBT	Follow the Mediterranean diet adapted for Latino cultures Latin American recipes altered by the Latina project RD to conform to the *¡Viva Bien!* diet Recipes from specific Latin American countries were modified using common staples to cover range of ethnic Latin American foods. Potluck dinners were key to the diet component Participants were shown how to modify their favorite recipes by incorporating the principles of the Mediterranean diet into their usual foods Colorful pamphlets were created in English and Spanish and included photographs of common Latino foods and Latina women
*En Balance*	Y	3	~ 7	Hispanic RDs, RNs, physicians, nutrition students	none	12	*En Balance* Diabetes Education Program	Classes were conducted in Spanish Recommendations followed culturally specific food groups, rather than forgoing traditional dishes The educators’ first language is Spanish. Therefore, they were able not only to communicate in Spanish without using translators but also to “connect” culturally study participants
*Latinos En Control*		24		lay leaders	usual care	20	Social Cognitive Theory	Intervention materials and activities tailored for the cultural and literacy needs of a low-income Latino population

1 Intervention referred to by their nickname or study PMID when nickname was not available.

Empty cells indicate information was not reported by the researchers in article nor previously published protocol. AADE, American Association of Diabetes Educators; CDE, certified diabetes educator; CHW, Community Health Worker; DSME, diabetes self-management education; EBT, evidence based treatment; HA, Hispanic Americans; kcal, calorie; MA, medical assistant; min, minute; MLP, Mediterranean Lifestyle Program; NCM, nurse case manager; PA, physical activity; PR, Puerto Rican; RD, registered dietitian; RN, registered nurse; T2D, type 2 diabetes; Y, yes.

Descriptions of the counselors carrying out the intervention and level of detail about the intervention itself ranged widely across studies. Counselors were referred to as “educator/facilitator” or “lifestyle community coach” with no further specifications in two studies. Six studies reported using counselors with no background in healthcare, such as lay leaders, *promotaras*, and Community Health Workers (CHWs). Of these, two reported their counselors had diabetes or had a close family member/friend with diabetes (*Imagínate una Buena Salud* and *Amigos en Salud*), and four studies had bilingual/bicultural counselors (11, 28, 30, 33). Seven studies had teams of counselors who were exclusively healthcare workers and three had teams that combined healthcare workers and layleaders. Registered dietitian nutritionists (RDNs) were reported to be part of six of these counseling teams and part of the training/coordinating team of three additional interventions (12, 16, 26). The training or use of motivational interviewing (MI), a patient-centered, goal-oriented counseling technique recently adopted in the dietetic profession (37), was reported in a total of five studies (11, 12, 14, 16, 26).

In terms of intervention frameworks and components, Social Cognitive Theory (SCT) was reported to be used in five interventions, and other models included Social Learning Theory, the Transtheoretical Model, Behavior Change Theory, and Self Determination Theory. Furthermore, all interventions mentioned the use of culturally tailored components, although the descriptions of these components ranged extensively. Whereas some interventions simply stated they used culturally relevant tools (14, 16, 30), other interventions extensively elaborated their cultural considerations (32). Most of the culturally tailored components mentioned were adapting and providing culturally relevant recipes, availability of the material in Spanish, and encouragement of family members or close friends to participate as a support person.

Finally, although very few studies reported the number of sessions/h dedicated to nutrition education/counseling, most of the interventions provided a description of their nutrition component. We summarized a total of nine nutrition topics and strategies commonly used throughout the included interventions in Table 3. Only eight out of the 18 primary studies (44%) reported using three or more of these nutrition components. The most popular nutrition education topics included promoting a food labels, healthy dietary pattern, and portion control; as they were mentioned in seven, six, and five interventions, respectively. Four studies explicitly mentioned carbohydrate monitoring/elucidating the effects of macronutrients on human physiology (12, 16, 19, 31). Encouragement for reducing consumption of “low-fat” foods was reported in five studies (18, 19, 22), but only two distinguished between the different types of dietary fat (16, 26). Promotion of dietary fiber was mentioned in three studies (16, 19, 26). Three interventions mentioned carrying out cooking demonstrations (22, 32, 33), three mentioned taking a trip to the grocery store (16, 21, 32), and one reported using food models and hand measurements (34). Finally, despite the large number of studies stating to use behavior change models, only two interventions specifically mentioned the use of goal setting as part of their nutrition intervention (13, 32). Short descriptions based on the information reported by each intervention were summarized in Supplementary Table S1.

**Table 3:** Checklist of the nine most common nutrition components of included interventions.

Intervention name	Nutrition component checklist									
	Portion control	CHO & MACRO monitoring	Low-fat	Dietary fiber	Goal setting	Healthy diet pattern	Food labels	Cooking demos	Grocery store trip	Sum
*Imagínate una Buena Salud*										
IMB-DSC	x	x					x			3
*EnForma-Diabetes*					x					1
Cariño							x			1
*DIALBEST*	x	x	x	x			x		x	6
MATCH										
28447545			x				x			2
26957533						x				1
28503930		x	x	x						3
*Unidas por la Vida*	x					x				2
*Amigos en Salud*										
Si, Yo Puedo Controlar Mi Diabetes!		x								1
15946117					x		x	x	x	4
La Diabetes y La Unión Familiar						x				1
21462725							x	x	x	3
*¡Viva Bien!*			x			x		x		3
*En Balance*	x					x	x			3
*Latinos En Control*	x		x	x						3

Presence of nutrition component is marked by “x.” Empty cells indicate information was not reported by the researchers in article nor previously published protocol.

### 3.4 Nutrition intervention impact and outcomes

Lastly, the reported dietary assessment tools and the general direction of dietary outcomes from each of the included studies are summarized in Table 4. Half out of the 18 primary studies used subscales of the Summary of Diabetes Self-Care Activities (SDSCA) measure, the Behavioral Risk Factor Surveillance System BRFSS, self-developed questionnaires, or did not report their assessment tool. The dietary assessment tools used by the other half of the interventions included short dietary screeners, 24-h recalls, and food frequency questionnaires (FFQs). Only two interventions reported using dietary assessment tools validated in a Mexican American group (19, 24).

**Table 4:** Nutrition assessment tools and nutrition related outcomes.

Intervention name	Dietary assessment tool						Summarized dietary outcome
	BRFSS	24-h recall	FFQ	SDSCA subscale	Screener	Other	
*Imagínate una Buena Salud*				x			↓ Days of consuming high fat foods in the previous week
IMB-DSC				x			↑ Food label reading ↑ Diet adherence
*EnForma-Diabetes*					14-item PREDIMED		↑ Mean Med-diet score ↓ Carbonated and/or SSB consumed per day ↑ Servings of fish/seafood consumed per week ↑ Legumes and nut consumption per week
Cariño	x						Study 1: ~ in FVs intake Study 2: ~ acculturation level and FV intake
*DIALBEST*		1				Food label questionnaire	Study 1: none Study 2: ↑ Food label @ 3,12,18 months Food label use mediated diet quality reduction in mean HbA1c levels ↓ HbA1c values with respect to ↑ diet quality
MATCH				x			~ change to diet intake
28447545					Rapid Food		~ change to diet intake
26957533	x						~ change to diet intake
28503930					Dietary Screener for Mexican Americans		~ Difference in fiber/fat intake between interventions ~ Correlations of mean fat and fiber intakes with A1C
*Unidas por la Vida*					Block “Alive”		↓ average daily GL, ↓SFA intake ↓ Fruit Intake ~ Vegetable Intake
*Amigos en Salud*	x						↑ proportion of patients consuming 2+ servings of FVs daily ↓ Intake of fatty foods ~ Mediation of diet in BMI decrease
Si, Yo Puedo Controlar Mi Diabetes!						Diabetes self-care scale developed by authors	↑ Days of following a healthful eating plan (from 3 to 6 days per week) ↑ Eating 5 servings of FVs per day (from 3 to 5 days) ↓ Eating high-fat foods, such as red meat and dairy (decrease from 3 to 2 days)
15946117		2					~ Dietary intake components (total KCAL, total fat, SFA, total CHO, fiber)
La Diabetes y La Unión Familiar						KABB	↓ Frequency of sweetened drink consumption (not carbonated soft drinks) ~ Frequency of FVs, soft drinks, or low- and nonfat milk consumption
21462725							~ Dietary Change
*¡Viva Bien!*			Patterson 1999				Study 1: ↓ % calories from fat Anglo orientation ↑ improvements in dietary supportive resources Latina orientation ↓ improvements in dietary supportive resources Study 2: Latina orientation ↓ percent calories from SFA U.S.-born women consumed ↑ SFA at baseline than foreign-born women Study 3: ↑ problem solving, self-efficacy, and UCLA-measured social support correlated with ↓ SFA intake ↓ SFA intake significantly correlated with ↓ HbA1c
*En Balance*			Southwestern				Study 1: NA Study 2: ↓ Dietary Cholesterol intake ~ Change in 15 other dietary measures Study 3: ↓ Dietary Cholesterol and total fat intake ~ Change in 27 other dietary measures ↓ VitC intake in women ↓ serum A1C were positively correlated to ↓% CAL from SFA ↓ total cholesterol values negatively correlated to changes in Ca, P, Zn, VitA, VitC intake ↓ serum LDL negatively correlated to changes in Ca, P, Zn, VitA, VitC, AA, energy, LA intake
*Latinos En Control*		3					Study 1: ↑ AHEI score ↓ KCAL, % fat, % SFA ~ % CHO Study 2: ↑ GI associated with ↑ HbA1c levels and WC

Dietary assessment tool chosen by intervention indicated by “x”; number of 24-h recalls administered and names for FFQ, Screener, and “Other” specified. Arrows indicate signficant difference between intervention and control group (↑: increase, ↓: decrease; ~: no significant difference). Reporting changes at the end of the intervention only. Empty cells indicate information was not reported by the researchers in article nor previously published protocol. AA, arachidonic acid; Ca, Calcium; FV, Fruit and Vegetable; kcal, calories; LA, linolenic acid; ns, not significant; P, Phosphorus; VitA, Vitamin A; VitC, vitamin C; SSB, sugar-sweetened beverages; WC, Waist Circumference; Zn, Zinc.

Dietary outcomes widely vary and cannot be easily compared since they were captured by different assessment tools. The SDSCA subscales’ and BRFSS’s scope was limited to capturing frequency of fruits and vegetables (FVs) and fatty food intake frequency (11, 14, 30). The studies using screeners captured additional information depending on the researcher’s nutrition components of interest: *EnForma-Diabetes* used the PREDIMED screener to measure changes in a Mediterranean dietary pattern and intake of SSBs, seafood, and pulses; the researchers using the Screener for Mexican Americans (19) focused on capturing dietary fiber and fat intake; and *Unidas por la Vida* used the Block Alive screener to capture glycemic load as well as FV and saturated fat (SFA) intake. Finally, researchers using 24-h recalls or FFQs could take a wider range of dietary components (total energy, macronutrients, micronutrients) (24, 32) and calculate overall diet quality scores (16, 26). *¡Viva Bien!* and *Dialbest* used 24-h recall and FFQ as their assessment tools respectively, but only reported diet quality and SFA intake, respectively.

Changes in HbA1c and one or more anthropometric measures (body weight, waist circumference, or BMI) are reported in Supplementary Table S2. These outcomes were reported in 88 and 72% of the primary interventions, respectively. Changes in HbA1c ranged from −1.4 to +1.2%, where significant changes ranged from −0.3 to −1.4%. Of note, changes in HbA1c ranged from −0.3 to −0.93% in the four interventions in which RDNs were part of the counseling team. Additionally, the three interventions with the highest significant change in HbA1c (−0.85 to −1.22%) and a nutrition intervention description reported using 3,4, and 6 of the 9 nutrition topics reported in Table 3. In terms of anthropometric measures, changes in body weight captured in 5 studies ranged from −2.3 to +0.6 kg; changes in waist circumference captured in 5 studies ranged from −0.98 to −4.2 cm; and changes in BMI captured in 7 studies ranged from −2.04 to +0.6 kg/m^2^. Interestingly, six interventions studied correlation or mediation effects between diet and clinical outcomes, where notably *Dialbest* reported food label use mediated reduction in mean HbA1c levels and decreases in SFA intake significantly correlated with decreases in HbA1c in *¡Viva Bien!* and *En Balance.*

## 4 Discussion

**Figure 2:**
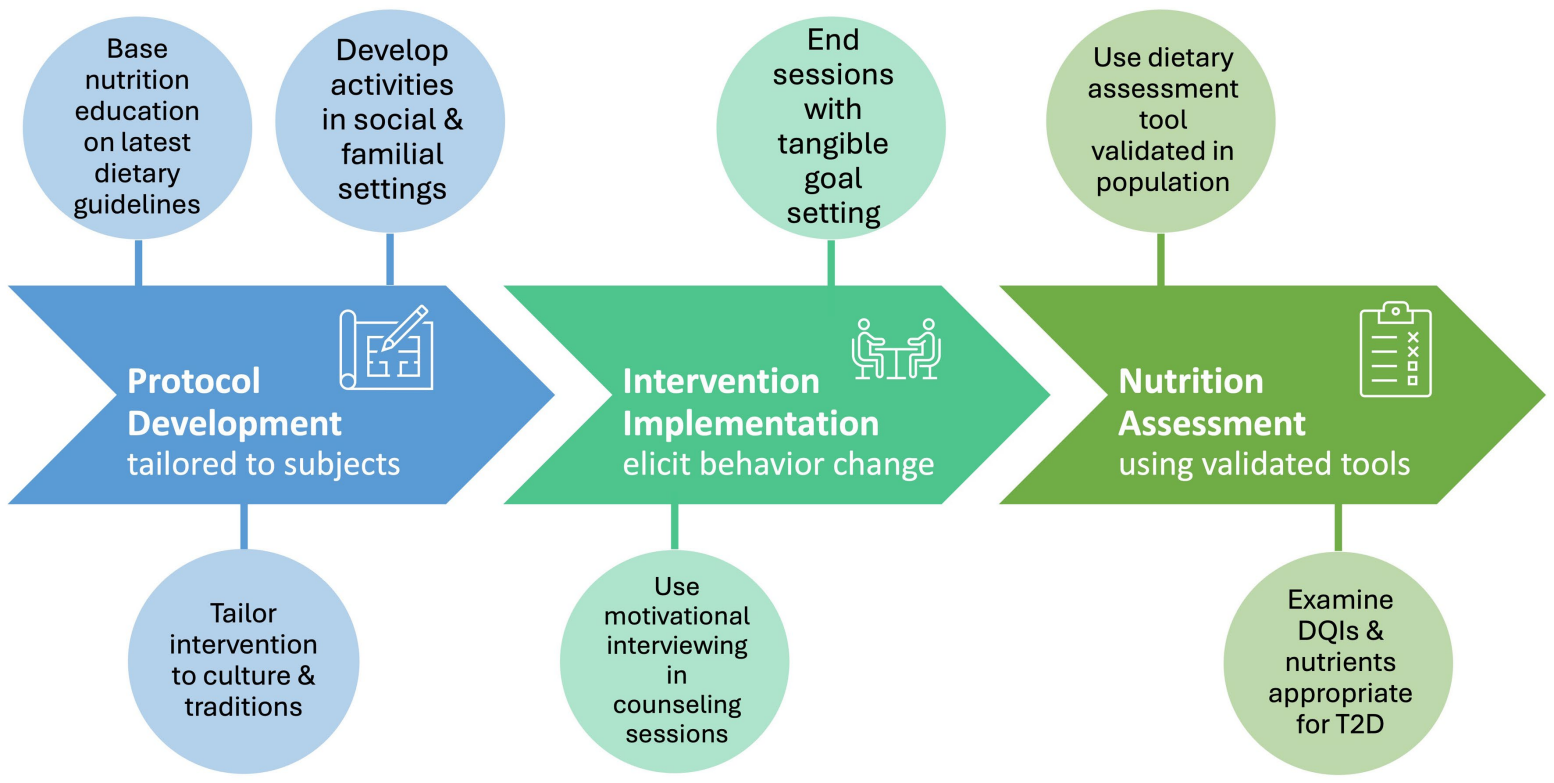
Summarized guide for developing effective nutrition interventions in US-based Latino/Hispanic communities with type 2 diabetes. DQI, Dietary Quality Indices.

The present review summarized the current evidence in the development and impact of nutrition-related components on diabetes self-management interventions in US-living Hispanic/Latinos. We described the characteristics of the participants, rigorously searched for pertinent intervention details in the primary analyses/supplemental information/published protocols whenever available, and thoroughly examined the nutrition assessment tools and dietary outcomes presented in each intervention. We observed a widespread adoption of culturally tailored strategies and nutrition-related components in the interventions studied. However, it is essential for future research to acknowledge several limitations and inconsistencies identified in our findings related to protocol development, intervention implementation, and nutrition assessment (Figure 2).

The level of information provided on the intervention’s content, development, and execution ranged quite extensively between studies. For example, although all researchers claimed their intervention was culturally tailored, reports on how these interventions were adapted to the Latino community ranged from simply “providing culturally relevant resources” to descriptions of many culturally relevant practices (e.g., culturally relevant resources, ethnic appropriate recipes, socializing and family-oriented settings). Tailoring individual nutrition needs on personal and cultural preferences is an essential goal of nutrition therapy (5). A recent systematic review assessing the effectiveness of culturally tailored diabetes prevention strategies in several minority populations found that interventions were most effective when they incorporated culturally relevant targeting strategies in the four domains of facilitators, language, location, and messaging (FiLLM conceptual framework) (38). Given the diversity of cuisines and cultures within the US Latino population itself, tailoring nutrition interventions to specific cultural practices and preferences is crucial for their effectiveness, and transparency in reporting these culturally adapted strategies is essential for the validity and reproducibility of the research. Varying levels of detail were also found for the description of the nutrition component of the intervention, ranging from simply stating its presence to elaborating a detailed description for each nutrition session. It is consequently difficult to compare the nature of the interventions between the different studies due to this lack of consistent reporting. There is a pressing need for greater emphasis on standardizing the description of interventions to ensure a level of detail that facilitates comparison, replication, and compliance with the latest dietary and DSME/S guidelines (Figure 2, Protocol Development).

This review underscores the importance for researchers to meticulously consider their selection of dietary assessment methods during protocol development (Figure 2, Nutrition Assessment). Utilizing a tool that is not only validated for dietary assessment but also tailored to the specific population and aligned with the intervention outcomes is crucial for accurately capturing dietary intake. The BRFSS is a surveillance system created by the CDC to track state and national level data on fruit and vegetable intake, and consequently may not be the most ideal tool to capture individual changes of overall dietary pattern (39, 40). Significant correlations of the SDSCA subscales with 3- or 4-day food records and FFQs were previously demonstrated, which suggest the SDSCA is an adequate tool to approximate general and specific diet (41). However, the Spanish version of the SDSCA was validated in a Hispanic community based in Spain (42), which is not representative of the varied Latino ethnicity subgroups present in the US. The use of FFQs has been validated to assess food intake during dietary interventions studies (43), however the use of an FFQ developed for the general population likely results in biased estimates if applied in minority populations, as demonstrated by Tucket *et al* in Hispanic adults (44). *EnBalance* demonstrated a good example of examining detailed changes in dietary intake using the Southwestern FFQ, which was previously validated for the Hispanic population (45). RDN-administered 24-h recalls, although high in participant and researcher burden (46), can help accurately capture complete dietary intake by inclusion of ethnic foods not captured in FFQs and overcome issues of participant literacy reported by several interventions (16, 26, 29). Additionally, they can also be used when a dietary assessment tool validated in the target population is not available. Investigating the dietary habits of the Hispanic/Latino population requires meticulous attention to cultural factors. Incorporating cultural adaptation into dietary assessment tools is imperative to ensure the accuracy and reliability of dietary intake evaluations within this ethnic group.

In addition to cultural adaptation, the selection of an appropriate dietary assessment method should prioritize monitoring dietary habits relevant to the management of T2D. The latest nutrition therapy goals set forth by the ADA (2019) emphasize the support of healthful eating patterns and nutrient dense foods in appropriate portion sizes balanced in macronutrients and reduced in alcohol, sodium, and nonnutritive sweeteners (47). It may therefore be preferable to prioritize assessment of overall diet intake over reports of a single nutrient or food group intake. The use of appropriate diet quality indices (DQI) should be considered in future interventions to approximate overall diet quality, as they are easily reproducible and comparable, analytically simple to compute, and result in meaningful, interpretable measures that can be associated with health outcomes (48) (Figure 2, Nutrition Assessment). Furthermore, a recent meta-analysis demonstrated that DQIs were effective at measuring change in diet quality in adults with and without chronic health conditions participating in RCTs with a dietary intervention (49). The Alternative Healthy Eating Index (AHEI) score, one of the validated DQI’s from the meta-analysis, was used in *Latinos en Control* to accurately capture participant dietary patterns from three administered 24-h recalls. This intervention successfully captured both general dietary patterns and intakes of specific nutrients in their primary analysis (26) and the researchers were consequently able to run interesting associations between dietary and anthropometric outcomes in their subsequent secondary analysis (27). The intervention carried out by *Latino en Control* exemplifies proper selection of nutrition assessment methods and outcomes for future interventions.

Finally, RDNs are an integral part of both the development of these interventions and overall T2D prevention and management strategies. The Academy of Nutrition and Dietetics have accumulated compelling evidence supporting MNT’s effectiveness in the management of several chronic conditions, including obesity and T2D (50, 51). Additionally, RDNs receive extensive training in applying MI in patient counseling sessions, which promotes the use of compassion and collaboration to illicit patient internal motivation and instigate sustainable behavior change (52) (Figure 2, Intervention Implementation). RDNs who are part of the US Latino community or have familiarity with its culture and culinary traditions are uniquely positioned to tailor MI, nutritional counseling, and other strategies effectively to this group. Indeed, the use of culturally targeted facilitators, individuals from the same cultural and social background as the targeted group, has been shown to lead to successful diabetes prevention interventions in several ethnic minority groups, including Hispanic/Latino population (38). However, given the shortage of bicultural healthcare professionals, ensuring that non-Latino/Hispanic providers are familiar and competent to provide socially oriented recommendations is equally important. Culturally competent care of RDNs and the rest of primary care team is fundamental for successful interventions by promoting patient trust, adherence to treatment plans, and ultimately reducing health disparities among minority groups (53, 54). This practice is particularly important in diabetes care, which requires individualized treatment plans and nutrition strategies that must be tailored to personal and cultural preferences, literacy and numeracy, access to healthful food choices, and ability to make behavioral changes of the target population (5).

## 5 Conclusion

This review describes the nutrition components of interventions aimed to improve T2D outcomes in US-based Latinos/Hispanics. Our results highlight the importance of selecting cultural adapted dietary assessment tools monitoring dietary habits relevant to the management of T2D. The inclusion of bicultural RDNs in the counseling team assures the promotion of culturally tailored nutrition recommendations delivered with methods facilitating behavior change to promote a successful nutrition intervention. Figure 2 aims to guide investigators in developing effective nutrition interventions for the Latino/Hispanic community with T2D, while also ensuring their methods and findings are clearly reported to the broader research community. These guidelines could be applied to other populations as well. Standardizing these practices will aid in facilitating intervention comparability and replicability, ultimately leading to improved health outcomes in this high-risk population.
