# Supplementary material for: A nutrition-focused review of the interventions in US-living Latino communities with type II diabetes
Source: Front Nutr. 2024 Sep 18;11:1418683. doi: 10.3389/fnut.2024.1418683 (PMC11445132; doi:10.3389/fnut.2024.1418683)
Supplement: Supplementary file 1 [file Table_1.DOCX]

**Supplementary Material**

| **Intervention Name** | **Nutrition Component Description** |
| --- | --- |
| Imagínate una Buena Salud |  |
| IMB-DSC | Information session:  • Monitoring carbohydrate intake  • Portion size control  • Intervals between/spacing of meals (several small meals) Behavioral skills: • Identify carbohydrates content of foods  • Read food labels for serving size and carbohydrate counts  • Control carbohydrate portion size |
| EnForma-Diabetes | • Session 1: administered a dietary questionnaire to guide counseling and reviewed the dietary content (oils, dressings, nuts, fish, and meats) and tailored co-developed action plan • Session 2: Similar format addressing dietary information related to beverages, desserts, fruits and vegetables, grains, and beans |
| Cariño | • 6 nutrition education group sessions (2 on food labeling) |
| DIALBEST | Nutrition Topics: • Food labels, portion size, grocery shopping • Carbohydrates, protein, sodium, cholesterol, saturated fat, trans fatty acid, and fiber • Impact of various food items on blood sugar levels and lipid profiles • Food insecurity among Latinos • Macronutrients – absorption, and digestion • Nutrition medical therapy for diabetes |
| MATCH |  |
| 28447545 | • Activities in the nutrition module included label reading and identifying healthy foods from fast food menus • *Promotoras* provided informational support for interpreting nutritional information from fast food menus and appraisal support for selection of low-fat foods |
| 26957533 | • Exercise and Food • Eating healthy • Blood sugar levels and glucometers • Taking care of your body |
| 28503930 | • Described the effect of high- and low-fiber foods and high- and low-fat foods on blood glucose diet • Focused on the benefits of consuming mainly plant-based foods • Describe family, cultural, and religious practices that affect food choices and habits |
| Unidas por la Vida | • Promoted reduction in caloric intake (1,200 –1,800 kcal/day) and an increase in caloric expenditure through moderate physical activity (150 min/week) • Participants were encouraged to self-monitor their daily intake of fruits and vegetables, protein, and carbohydrates using “Create Your Plate” guidelines |
| Amigos en Salud |  |
| Si, Yo Puedo Controlar Mi Diabetes! | • Relationship between carbohydrate intake and blood glucose levels  • Menu planning  • Food measurements |
| 15946117 | • Dietary guidelines • Self-regulation: role of diet, physical activity, medications  • Menu planning • Supermarket tour • Group cooking and cooking demonstrations  • Group meals with guided group discussions  • Label reading  • Use of measuring aids  • Goal setting (group and individual) |
| La Diabetes y La Unión Familiar | • Provided information on food choices and physical activity so families can make informed choices • Key concepts related to diet, exercise, and family support are introduced and discussed using pictorial flipcharts, educational games, food sampling and preparation, and low-level physical activities. • *Ser Saludable* (Being Healthy) session addresses the relationship between physical activity, food choices, and diabetes control and prevention |
| 21462725 | Education sessions: • Dietary Principles for Mexican American foods • Food preparation • Food labels (trip to grocery store) |
| ¡Viva Bien! | • Encouraged participants to follow the Mediterranean diet adapted for Latino nationality subgroups • Emphasizing vegetables, fruits, legumes, nuts, cereals, olive oil, limited animal fat, and portion control. • The goal was to lower the fat and calories while maintaining flavor with traditional ingredients and spices.  • The Latina dietitian conducted cooking demonstrations to show new methods for preparing typical foods  • Participants were shown how to modify their favorite recipes by incorporating the principles of the Mediterranean diet into their usual foods |
| En Balance | Nutrition Classes: • The Role of Nutrition in Diabetes Care • How to Choose Healthier Foods • Nutrition Labeling • Weight Control Principles • Dining Out • Food Portions Control • Food Pyramid • Recommendations for changes in diet focused on smaller portion sizes and choosing healthier alternatives • Nutrition topics were taught using a hands-on, using food models and comparable hand measurements • En Balance Diabetes Education Program is a hands-on, culturally competent diabetes education program for Hispanics. All participants received free glucose monitors, strips, and lancets |
| Latinos En Control | • Specific dietary targets of the intervention included decreasing intake and portion size of high glycemic index foods (e.g., starchy vegetables), sodium and saturated fat intake, and increasing fiber intake |

**Supplemental Table 1. Short description of nutrition interventions of included interventions.**

| **Intervention Name** | **HbA1c (%)** | **BW (kg)** | **WC (cm)** | **BMI** |
| --- | --- | --- | --- | --- |
| Imagínate una Buena Salud | − 0.27 (ns) |  | −0.98 (ns) |  |
| IMB-DSC | - 0.48 |  |  |  |
| EnForma-Diabetes | + 0.2 (ns) | 0.0 (ns) |  |  |
| Cariño | -0.51^1^ |  |  | - 0.3 (ns) |
| DIALBEST | -0.93 | + 0.6 (ns) |  |  |
| MATCH | -0.69^1^ | - 2.3 |  |  |
| 28447545 | -0.8 (ns) |  |  |  |
| 26957533 | -0.8 |  |  |  |
| 28503930 | -1.22 |  | -2.71 (ns) | 0.01 (ns) |
| Unidas por la Vida |  | - 0.7^1^ |  |  |
| Amigos en Salud | -1.4 |  |  | -0.5 (ns) |
| Si, Yo Puedo Controlar Mi Diabetes! | -0.3 (median) |  |  |  |
| 15946117 | -0.85 |  | -4.2 (ns) | -0.10 (ns) |
| La Diabetes y La Unión Familiar |  |  |  |  |
| 21462725 | +1.2 (ns) |  |  | −.6 (ns) |
| ¡Viva Bien! | 0.0 (ns) |  |  | + 0.6 (ns) |
| En Balance | − 0.57 (ns) | - 0.41 (ns) | − 0.15 (ns) | - 2.04 |
| Latinos En Control | -0.88 (ns) |  |  |  |

**Supplemental Table 2. Reported changes in HbA1c and anthropometric-related outcomes after selected interventions.**

^1^Not absolute change, rather % lower in the CHW group compared to usual care from regression models. Empty cells indicate information was not reported by the researchers in article nor previously published protocol. Abbreviations: BW: Body Weight; ns: not signficant; WC: Waist Circumference.
